# Unmet support needs of early-onset dementia family caregivers: a mixed-design study

**DOI:** 10.1186/s12912-014-0049-3

**Published:** 2014-12-19

**Authors:** Francine Ducharme, Marie-Jeanne Kergoat, Renée Coulombe, Louise Lévesque, Pascal Antoine, Florence Pasquier

**Affiliations:** Faculty of Nursing, Université de Montréal, 2375 Côte Ste-Catherine, Montréal, Quebec H3T 1A8 Canada; Research Centre, Institut universitaire de gériatrie de Montréal, 4565 Queen-Mary Road, Montreal, Quebec H3W 1W5 Canada; Specialized Medicine Department, Institut universitaire de gériatrie de Montréal, 4565 Queen-Mary Road, Montreal, Quebec H3W 1W5 Canada; Memory Clinic, Institut universitaire de gériatrie de Montréal, 4565 Queen-Mary Road, Montreal, Quebec H3W 1W5 Canada; Psychopathology and Health Psychology, Université de Lille 3, Lille, France; Centre National de référence pour les patients jeunes atteints de la maladie d’Alzheimer et maladies apparentées, Université de Lille, Lille, France

**Keywords:** Caregivers, Early-onset dementia, Unmet support needs, Partnership approach, Professional interventions

## Abstract

**Background:**

Though advances in knowledge and diagnostics make it possible today to identify persons with early-onset dementia or a related cognitive disorder much sooner, little is known about the support needs of the family caregivers of these persons. The aim of this study was to document the unmet support needs of this specific group of caregivers. This knowledge is essential to open avenues for the development of innovative interventions and professional services tailored to their specific needs.

**Methods:**

This study was conducted using a mixed research design. Participants were 32 family caregivers in their 50s recruited through memory clinics and Alzheimer Societies in Quebec (Canada). The Family Caregivers Support Agreement (FCSA) tool, based on a partnership approach between caregiver and assessor, was used to collect data in the course of a semi-structured interview, combined with open-ended questions.

**Results:**

The unmet support needs reported by nearly 70% of the caregivers were primarily of a psycho-educational nature. Caregivers wished primarily: (1) to receive more information on available help and financial resources; (2) to have their relatives feel valued as persons and to offer them stimulating activities adjusted to their residual abilities; (3) to reduce stress stemming from their caregiver role assumed at an early age and to have the chance to enjoy more time for themselves; and (4) to receive help at the right time and for the help to be tailored to their situation of caregiver of a young person.

**Conclusions:**

Results show numerous unmet support needs, including some specific to this group of family caregivers. Use of the FCSA tool allowed accurately assessing the needs that emerged from mutual exchanges. Avenues for professional innovative interventions are proposed.

## Background

It has been well documented that the onset of dementia in persons 65 years old or over constitutes a particularly painful event for both the sufferers and their family caregivers. However, much less is known about the repercussions on the families of young persons with the disease [1,2], even though advances in knowledge and diagnostics now allow identifying much sooner cases of early-onset dementia, that is, those involving persons under 65 years of age [3]. The fact that little research has been conducted on early-onset dementia has been attributed in part to the fact that dementia is associated with old age [4] and that it is difficult for young persons and their families, as well as for healthcare professionals, to envisage this disease occurring at a young age.

The most common types of early-onset dementia are Alzheimer’s disease, which is often associated with a genetic etiology, followed by frontotemporal degeneration [5]. However, what often sets these types of dementia apart from late-onset types is the saliency of behavioural, praxic, executive and language problems over memory loss [1,6]. Moreover, the delay between the appearance of the first signs of dementia and diagnosis has been found to be longer for early-onset forms, particularly on account of lay representations that associate dementia with old age [7].

The context of early-onset dementia, too, is very particular. It is often characterized by spouses in their 50s, with children at home, and at the height of their working life [5]. Their marital life is upset by the advent of dementia at a time when such an occurrence is supposed to be highly unlikely [1]. The cognitive impairments of the sufferer and the person’s diminished ability to perform daily tasks lead to a growing dependence that forces people to redefine their spousal identity and relationship. Interpersonal relationships are necessarily restructured as the cared-for person becomes more and more dependent on family to meet his or her needs. This restructuring is extremely demanding the less reciprocal the relationships become [1,8]. Moreover, the caregivers of younger sufferers report higher levels of burden and stress than do the caregivers of older sufferers [1,9,10]. Lastly, it has been reported [11] that couples often face financial difficulties stemming from loss or reduction of employment.

Among the very few studies to examine the lived experience of caregivers of younger sufferers, the one by Ducharme and colleagues [12] allowed documenting that the daily reality of spouse family caregivers was marked primarily by the following: long quest for diagnosis; denial of diagnosis and non-disclosure to others; difficulty managing behavioural and psychological symptoms; grief over loss of spouse, married life and midlife projects; difficulties associated with assuming caregiver role prematurely and juggling this with other roles; and difficulties planning for the future.

In light of the growth in diagnosed cases of Alzheimer’s or related dementias at an increasingly younger age, healthcare professionals, particularly nurses working in primary care or in cognition clinics, are more frequently in contact with families living with this situation. In this regard, it has been demonstrated that healthcare professionals know little about the support needs of this particular group of caregivers and, consequently, are at a loss how to help them and improve their quality of life [13]. These observations bring to the fore the necessity of documenting the support needs of these caregivers who, to our knowledge, have not been a specific focus of research to date. This is all the more important in that, according to the caregiving models developed by Pearlin, Mullan, Semple, and Skaff [14] and by Schulz and colleagues [15], informal and formal support could have a direct or a mediating effect on caregiver well-being.

Against this background, we undertook a study to document the unmet needs for support of early-onset dementia caregivers in the aim of opening avenues for the development of nursing interventions and professional services tailored to their needs.

### Conceptual underpinnings

The study was based on a partnership approach with participating caregivers. Such partnerships rest on meaningful, non-prescriptive dialogue respectful of the viewpoints of all stakeholders [16,17]. In this context, interactive exchanges allow partners to be considered co-experts [17-19]. In our study, the family caregivers–the leading actors in their daily reality–were thus considered experts of their own support needs.

The relevance of working in partnership has been recognized in various health disciplines. In nursing, in particular, authors feel partnership based on an alliance with service users to be at the very heart of the discipline [20-22]. Furthermore, this type of approach is believed to foster empowerment by recognizing the ability of the actors to identify their own needs [19,20]. This was the approach chosen to investigate the support needs of family caregivers of younger persons with Alzheimer’s or a related dementia.

## Methods

### Design

We used a mixed research design combining a quantitative and a qualitative approach to document the unmet support needs of family caregivers in partnership with them.

### Setting and participants

Family caregivers were recruited via fifteen sites, mostly memory clinics and Alzheimer Societies in Quebec, Canada. Participants were the spouse or an offspring self-defined as the person principally responsible (notion of primary caregiver) for a person diagnosed with Alzheimer’s or a related dementia before age 65 by a neurologist, psychiatrist or geriatrician.

A designated professional was mandated in each clinical facility to contact potential participants. If these persons gave their consent, the professional forwarded their contact information to the project coordinator. The coordinator then called them to explain the project and solicit their participation. In all, 32 caregivers took part in the study. This number was determined by data saturation, that is, by thematic redundancy in caregiver perception of their unmet support needs [23].

### Data collection tool

The Family Caregivers Support Agreement (FCSA) tool was used to document the support needs of caregivers. This tool, as its name suggests, is consistent with our frame of reference and a partnership approach. The FCSA tool derives from the validation, in the Canadian context, of the Carer’s Outcomes Agreement Tool (COAT) developed by British and Swedish nurse researchers and presently implemented in various Swedish settings [24]. The COAT is an innovative tool developed using a constructivist approach with the participation of caregivers, practitioners, and community groups [25] who, together, identified the support needs deemed essential to meet in order to improve caregiver quality of life.

Given the possible cultural differences not only in terms of language but also in how certain concepts are understood, the FCSA tool was subjected to a transcultural validation in order to adapt the original version of the COAT to the Canadian context [26,27]. This validation was conducted with English- and French-speaking family caregivers and healthcare providers working in homecare services [26]. Then, an ecological validation [28] was carried out by field-testing the tool with the participation of practitioners and caregivers and subsequently holding focus groups and individual interviews in order to refine and tailor the instrument [29].

Like the original COAT, the FCSA tool covers four dimensions. The first, *helping you care for your relative* (13 items), takes account of the different types of information and help that could be useful for the purpose of caring for the sick relative (e.g., information on available help and services). For each type of information and help presented, respondents indicate whether it would be useful for them, whether it is already being received, or whether the need does not apply to their situation. The second and third dimensions concern, respectively, help to make life better for the sick relative (8 items) and for the caregiver (9 items). The response scale allows respondents to indicate whether the proposed help would make life better for the relative or for them, whether it is already being received, or whether it does not apply to their situation. Each of these three dimensions is followed by open-ended questions asking respondents to make comments, identify other needs not covered by the tool, and identify other types of help that would be useful. As for the fourth and last dimension, *getting quality help* (8 items), it regards quality criteria that caregivers deem important to respect when it comes to help offered by services. Caregivers indicate whether the help and services they receive meet their expectations in terms of quality or whether they should be improved. Finally, an open-ended question completes this discussion, in which caregivers are asked to indicate whether other aspects of services could be improved. For caregivers presently receiving no services, the open-ended question was the following: “What do you expect of the help you wish to receive in terms of quality?”

### Data collection procedure

Ethics approval has been obtained from the Research Ethics Board of the *Institut universitaire de gériatrie de Montréal* (# 10-11-018). The Board’s mandate is to ensure that consent from research participants is obtained in compliance with the ethical standards stipulated by the Quebec government and Canada’s major funding agencies.

Data were collected through interviews with the family caregivers. At the start of the interview, participants had to sign a consent form. The interviews to complete the FCSA tool, which lasted 90 minutes on average, took place face to face with an interviewer trained by the research team on conducting qualitative interviews. The interviews were held at home, at the research centre or at the offices of a local Alzheimer Society, at the participant’s discretion. The interviews were digitally recorded.

### Data analysis

Descriptive statistics (means, standard deviations, and percentages) were calculated to draw a profile of the participants. In order to identify unmet needs, we ascertained the number and percentage of caregivers who reported an unmet need under each of the four dimensions covered by the FCSA tool. More specifically, the aim was to ascertain the number of caregivers who indicated needing more information or help in connection with caring for their sick relative, as well as the number of those who mentioned a need that could be met in order to make life better for their relative or for them. We also took account of the number of caregivers who wished to see services improved.

The responses given to the open-ended questions in each interview were transcribed verbatim. The data were subjected to a thematic content analysis based on the interactive data analysis model proposed by Miles and Huberman [30]. According to this model, data collection and data analysis activities constitute a continuous, interactive process that is complete upon achievement of data saturation. The content analysis was carried out by two members of the research team who independently read the transcripts of the first eight interviews. They then developed a coding scheme, flagging the statements deemed relevant to the main themes of the open-ended questions. Then, the two researchers carried out an inter-subjective validation by consensus of the coding scheme, which was used thereafter to conduct the thematic content analysis of the data subsequently collected.

## Results

### Caregivers’ sociodemographic profile

As shown in Table 1, the caregivers were mostly women (75%). Their mean age was 54.28 years (*SD* = 10.58) and their average number of years of schooling was 14 (*SD* = 3.31). The male caregivers were older (*X* = 57.13 years, *SD* = 8.79) than their female counterparts (*X* = 53.33 years, *SD* = 11.12). The caregivers were mainly the spouse (78%) of the person cared for. Some (28%) had children at home, more than half of which were less than 18 years old. Eighteen of the caregivers (56%) were gainfully employed, of which two-thirds (*n* =12) full-time. To help them deal with their work situation, these caregivers received respite services. In some cases, the cared-for relative was still able to stay alone during the day. Of the unemployed caregivers, 10/14 wished to continue working full-time, to work more, or to return to work.

**Table 1:** **Sociodemographic and descriptive characteristics of caregivers (** ***N =*** **32)**

**Variable**	***n*** **(%)**	**Mean (SD)**
Sex		
Female	24 (75)	
Male	8 (25)	
Age (years)		54.28 (10.5)
Years of education		14.34 (3.2)
Family relationship with relative		
Spouse	25 (78)	
Offspring	5 (16)	
Other	2 (6)	
Children at home	9 (28)	
Gainfully employed	18 (56)	
Household income (CAN$)		
Less than 30,000	5 (16)	
30,000 to 70,000	12 (38)	
More than 70,000	12 (37)	
Refused to answer	3 (9)	
Type of dementia		
Alzheimer	19 (60)	
Frontotemporal	8 (25)	
Other	5 (15)	
Months since diagnostic disclosure		36 (33.7)
Months since first signs and symptoms		69 (40.9)
Hours/week of caregiving		83.7 (61.4)
Receipt of formal services	24 (75)	

The diagnosis of dementia (60% Alzheimer-type dementia and 25% frontotemporal degeneration) had been disclosed on average 36 months earlier (*SD* = 33.7). However, according to the caregivers, signs and symptoms had appeared long before, on average 69 months prior to diagnosis (*SD* = 40.9), which is to say nearly 10 years earlier. The caregivers provided on average nearly 84 hours a week (*SD* = 61.4) of support, help and care to their relative. Twenty-four participants (75%) received services, including for housework, personal and nursing care, and respite. Given the caregivers’ young age, respite made it possible for them primarily to continue working.

### Unmet support needs

The results of the FCAS tool regarding the unmet support needs of family caregivers are presented according to the four dimensions covered by the tool and to the corresponding open-ended questions, and are illustrated through transcript excerpts. Tables 2 and 3 give the principal results.

**Table 2:** **Unmet support needs according to FCSA tool (** ***N*** **= 32)**

**Dimension**	**Need**	**Would be useful** ***n*** **(%)**
*1. Need to help you care for your relative*		
More information on:	Type of help available and how to get it	25 (78)
	Financial assistance available and how to apply for it	23 (72)
	Your relative’s illness and treatment	13 (41)
	How to adapt home better for providing care	13 (41)
	Who to contact in case of emergency	13 (41)
More help:	From your family and your relative’s family	20 (63)
	To learn skills you need to care for your relative	19 (59)
	With physical aspects of providing care	11 (34)
More help to talk openly		
about your care		
situation:	With your family or your relative’s family	17 (53)
	With other caregivers (e.g., when you feel lonely)	16 (50)
	About alternative solutions to homecare	16 (50)
	With your relative	14 (44)
	With your employer	1 (3)
*2. Need to make life better for your relative*		
	Make relative feel still valued as a person	23 (72)
	Offer relative fun and stimulating leisure activities	22 (69)
	Allow relative more contact with friends	20 (63)
	Allow relative more contact with own family and yours	17 (53)
	Have someone considerate to keep relative company and to talk to	16 (50)
	Allow relative to continue living at home	13 (41)
	Ensure relative is free of pain and discomfort	10 (31)
	Ensure relative feels clean and comfortable	9 (28)
*3. Need to make life better for you*		
	To be able to reduce the stress you feel	24 (75)
	To have more free time for activities you enjoy	22 (69)
	To be able to take a break or a holiday	19 (59)
	To be able to set your limits as a caregiver	19 (59)
	To be able to get a good night’s sleep	15 (47)
	To do fun things together with your relative	15 (47)
	To spend more time with your family	13 (41)
	To go on working	10 (31)
	If people around you (e.g., your relative, family, showed their appreciation for the care you provide friends)	9 (28)

**Table 3:** **Unmet needs regarding quality of help received (** ***N*** **= 24)**

**Dimension**	**Need**	**Could be better** ***n*** **(%)**
Getting quality help from the people providing help^a^		
	Arrive at the expected moment	9 (38)
	Respect your routines and ways of doing things as much as possible 7 (28)	
	Provided by same person or by people you know and trust	6 (25)
	Agree to have discussions with you and your relative	6 (25)
	Treat your relative and you with dignity and respect	3 (13)
	Value your knowledge and skills	3 (13)
	Get to know and care about your relative as a person	3 (13)
	Have the right knowledge and skills	2 (9)

^a^Eight (25%) caregivers were currently not receiving help.

#### Need to help you care for your relative

More than 70% of the caregivers would have liked to receive more information on the type of help and financial assistance available and how to get it (Table 2). For 41% of them, it would have been useful, also, to have more information on their relative’s disease and its treatment, ways to adapt their home to provide care, and persons to contact in case of emergency:

“*I find that not enough is done to publicize the services offered… We learn about them bit by bit! A complete list or directory of the resources in the region should be drawn up and distributed… It’s always up to us to dig things up, to scour the internet…*” (Caregiver (CG) #30).

They also would have liked information to help the family understand this disease that occurred at such an early age and to know how to disclose the diagnosis and its consequences to the immediate and extended family. The question of heredity, in particular, was often raised:

“*… give the extended family some info. My husband has two brothers. They’re afraid of this disease. Is it genetic? Hereditary?*” (CG #32).

In terms of help needed, caregivers would have liked more support from their family network (63%) and training in the skills needed to provide care (59%):

“*It’s hard for family and friends to realize that when I come home after dealing with problems all day at work, all I do is deal with more problems… I have no one to confide in anymore … no tenderness… no support … I’m lonely! … and all they (family and in-laws) have to say to me is ‘take care of yourself’…”* (CG #26).

Respondents (50%) reported needing more help to talk openly about their caregiving situation with their family and other caregivers, and about alternatives to homecare. The caregivers confided more precisely that they would have liked to receive help from the extended family rather than from their children, as the latter had “their life to live”.

Interestingly, fewer respondents, about one-third of them, indicated needing more help with the physical care of their relative (Table 2).

#### Need to make life better for your relative

Five of the eight needs under this dimension were reported as unmet by half of the respondents or more (Table 2). More precisely, nearly 70% of the caregivers reported that being valued as a person and having more stimulating activities would make life better for their relative:

“*…stimulating, fun activities to pass the time, that’s what’s missing so much …anything, outings just to get out of the house, picnics… Places for old folks, it’s not for him!*” (CG #13).

Other needs, too, were just as important to meet for the sake of the relative, such as having more contact with the social network, especially friends (reported by 63% of the caregivers), having a sympathetic ear to talk to (50%), and continuing to live at home (41%):

“*Someone to keep her company: that’s a big need! It would reassure me to know that my wife is not alone and that she has someone to interact with. Someone to stay with her at home and to engage her in stimulating activities…*” (CG #10).

#### Need to make life better for caregivers

One of the major unmet needs under this dimension was receiving support to reduce stress (75% of the caregivers):

“*How much further will I be able to go…? My husband moves around all over the place all day long, he touches everything, he can’t eat alone, he often chokes on his food…*” (CG #13).

“*You forget to take care of yourself when you’re a caregiver. You’re in it up to your neck, you’re completely consumed by the disease. That’s a mistake* [forgetting to take care of yourself]” (CG #2).

This need to reduce stress was directly related to other caregivers’needs, namely, to take a vacation and to set limits as caregivers (59%). Having more free time for oneself (69%), also, was reported by the vast majority of the family caregivers. Finally, given their age, some caregivers had to deal not only with their sick spouse but also with aging parents with diminished autonomy, a situation that entailed particular needs:

“*A lady to keep my mother, who has Alzheimer’s, company would leave me more time for my sick husband…*” (CG #32).

#### Getting quality help

This last dimension covered by the FCSA tool allowed documenting whether help and support currently received by the caregivers could be improved. As shown in Table 3, 75% of the caregivers received help and the most important improvement had to do with timeliness, that is, getting help in a timely fashion (38% of caregivers).

“*The waiting lists are long…. You get help but it’s rationed. You’re never sure of anything!*” (CG #4).

“*The community centre has 51 families waiting for services in our area. I feel fortunate for the help I get* [30 min/week] *when I think about that*” (CG #28).

“*People in their 50s are a band apart. My own aging parents get better services than we do* [caregiver and care recipient]” (CG #9)*.*

Moreover, one-quarter of the respondents underscored the importance of respecting their routines related to caring activities, having care provided by the same person or by someone known and trusted by the caregiver, and having an agreement between the caregiver, the person cared-for and the service provider regarding the type of care to offer.

As for the caregivers who had yet to receive any help (25%), the qualitative data showed that they were in one of the following four situation: a) at the very start of the help trajectory and did not manifest the need for help; b) on a waiting list for requested help on account of limited service availability; c) they requested no help on account of caregiver or care-recipient denial of the disease; or d) they were reluctant to request any help outside their informal support network.

Overall, the results allowed us to observe the presence of numerous unmet support needs under each of the dimensions covered by the FCSA tool in this specific group of family caregivers of younger persons with Alzheimer’s or a related dementia. In sum, four unmet support needs were reported by 70% or more of the caregivers. Two of these needs had to do with information concerning the type of help available, including financial assistance, and how to get it. One need regarded the relative’s quality of life, that is, to be valued as a person (ensuring his/her dignity) and another, the caregiver’s quality of life (to be able to reduce stress). Ultimately, 16 of 30 needs covered by the tool were unmet according to the perception of 50% of the caregivers.

In the next section, we discuss these results and propose interventions to improve the fit between these needs and services offered.

## Discussion

The aim of this study was to document unmet support needs among early-onset dementia family caregivers. To this end, we used a mixed research design and a partnership approach. Results evidenced that these caregivers, who for the most part were spouses in their 50s often with children at home, have numerous needs for support.

As mentioned above, aside from the fourth dimension regarding quality of help received, 16 of the 30 needs investigated under the first three dimensions covered by the FCSA tool were unmet in 50% or more of the caregivers. Of these needs, six were particularly prevalent, as they encompassed 69% or more of the respondents. It is not surprising that two of these prominent needs (need for more information on available help and financial resources) arose in numerous caregivers given the large number of hours of care per week (84 on average) that the caregivers offered their sick relative and the fact that many of the caregivers had to quit their gainful employment or cut their hours of work. Two other needs that emerged from our analysis concerned the relative’s quality of life. In the face of the very nature of the disease, the caregivers observed that making their relatives feel valued as persons and providing them with a greater number of stimulating activities would contribute to their well-being. Preserving the abilities of these young people is essential to maintaining their self-esteem, dignity and sense of usefulness. Oftentimes, young persons are fine physically and could put their residual abilities to good use in the context of activities better adapted to their age.

Regarding quality of life, a large number of caregivers would have liked to reduce the stress related to their role and enjoy more time for leisure activities. The caregivers interviewed are young, have full family, social and professional lives and, to top things off, must assume a caregiver role at an early age and grieve the losses associated with this situation, including the sudden change in their marital life. From the viewpoint of the theoretical model of caregiving developed by Pearlin and colleagues [14], the experience of caregivers can be explained by the fact that they are exposed to multiple stressors. Some of these are part of their daily life, notably: relational deprivation (e.g., being less able to confide in his/her relative), family conflicts (e.g., disagreement between caregiver and family members about the cared-for person’s disability and its seriousness), and work-caregiving conflicts (e.g., dilemmas and pressure stemming from demands of caregiving and employment). Role captivity is another source of stress under Pearlin’s model. This arises when caregivers feel chained to their caregiving responsibilies in the face of activities they must forego. Also. given their caregiving experience, it is not surprising that, besides wishing to live with less stress and to have more time for social activities, caregivers would like, in particular, to receive more help with how to talk openly with family and other caregivers about their caregiving situation and how to set their limits as caregivers.

It is interesting to note that in addition to being numerous, the participants’ needs are primarily psycho-educational needs rather than instrumental needs. The dominance of these needs can be explained easily if we consider that early-onset dementia family caregivers experience difficulties above all of a psychological nature, as reported in an earlier study [12]. The primacy of psycho-educational needs reflects the comprehensiveness and complexity of the caregiver role. This role goes far beyond the instrumentality associated with accomplishing care tasks. These tasks constitute only the tangible aspect of the caregiver role. There are also other aspects of caregiving that are much less visible. These, commonly referred to as its intangible aspects [31], lie at the heart of a relational process involving the caregiver, the sufferer, and the family. Indeed, caregiving by its very essence takes place within a relational process that cannot be divorced from the relational history of these partners in care. The intangible aspects include, in particular, what caregivers do to maintain a satisfactory relationship with their spouse or partner and to cope with the difficult emotions they feel every day. From this perspective, it would be more appropriate to say that the caregiver role consists of taking care rather than providing care. It is important to underline that our study revealed a lack of support in connection with the intangible aspects of care.

The last dimension that we investigated among caregivers concerned quality of services. Our results showed that caregivers who received help in the course of their trajectory were generally grateful for the services received even though they were not always delivered in a timely fashion. This situation can be explained, in part, by the long delay in obtaining a diagnosis and by the difficulty finding available resources in general, but also resources tailored to the specific needs of this group of younger family caregivers who are on a different schedule and at times still hold a job. In short, it seems that, once a diagnosis is established, the wait time to receive help is inadequate. The participants pointed out also that the support offered is not specific to their condition as evidenced by the striking comment by one participant to the effect that support was more easily available for caregivers of older persons than for those of persons in their 50s.

The results demonstrate, also, the relevance of assessing unmet needs for support in partnership with caregivers. Indeed, the caregivers expressed, within the context of our assessment, the wish that help be offered following discussions with service providers. These young caregivers belong to a generation in which most know that they have the right to take part in the decisions concerning the care and services that they should have access to. A partnership approach allows them to be heard, to feel empowered by obtaining recognition of the abilities they acquire day by day providing care, and to be considered co-experts capable of taking an active part in the assessment of their needs. According to a study conducted by Raivio and colleagues [32], nearly 69% of the dementia family caregivers in their study reported having no say in the services that they were offered, a result supporting the importance for their voices to be heard. The use of an instrument such as the FCSA tool, which is based on a partnership approach, proved fruitful if not indispensable for documenting the support needs of caregivers accurately and for gaining a better understanding of what it means to be an early-onset dementia caregiver.

Further, although this study was undertaken with a small number of participants, the fact remains that data saturation was achieved. What’s more, the observations made can already orient healthcare professionals, especially nurses, in their role with these caregivers of young dementia sufferers. In the present context where the primary determinant of the healthcare services available and offered is the state of health of the sufferer [33], it is essential that the needs of caregivers also be taken into account so that services target not only persons with a disease but also the caregiver-care-recipient dyad.

Like all other healthcare professionals, nurses have to be alert to the possibility that a young person can suffer from dementia and that such an event can constitute a crisis situation affecting the entire family system. Needless to say that one of the first measures to consider is suggesting to caregivers that they seek a physician’s diagnosis sooner rather than attributing a relative’s early signs and symptoms of disturbed behaviour to burnout or depression. This will avoid an overly long delay in obtaining a diagnosis of early-onset dementia.

Once a diagnosis is established, primary-care nurses have a key role to play in the systematic assessment of the support needs of this group of caregivers, a process that can contribute to reduce their uncertainty and, as mentioned earlier, their feeling of powerlessness [12]. In this regard, the FCSA is a tool with application potential in clinical practice, as the mutual agreement that underpins the instrument improves the chances of achieving a better fit between needs and support offered [15,34].

Also, despite the scarcity of resources for these family caregivers of young persons, it is imperative that they be made aware of those that exist and are available to them, such as respite services, daycare centres, Alzheimer Society services, and support groups. Though these younger caregivers generally have the capacity to seek the information that they need on the web, facilitating their access to this information or filtering it could be helpful in light of their busy schedules and the knowledge that they sometimes lack to be able to assess the quality of this information, particularly when it is of the medical sort.

Above all, innovative forms of support designed specifically for young-onset dementia caregivers are needed. In this regard, some interventions have been proposed in the literature in recent years that could allow meeting several of the needs identified in this study. These include assigning a nurse case manager to assess specific caregiver support needs at time of diagnostic disclosure and to ensure follow-up of the caregiver-care-recipient dyad across their care trajectory [35]. Also, opening a clinical file for caregivers would allow considering them as clients of healthcare services and recognizing the health risks associated with their role [13]. Moreover, various modalities (i.e., at home, by telephone, or online) of psycho-educational interventions to reduce stress [36] have been put forth, as have systemic family interventions [37]. New forms of respite, too, have been suggested to meet the needs of caregivers in the workforce who often still have children at home [36]. However, “real respite” and time allocated specifically to caregivers are just as essential for preserving their psychological and social health. In addition, daycare centres should be redesigned to offer stimulating activities in line with the residual abilities of young sufferers [13]. Given the possibility of diagnosing dementia sooner nowadays, emphasis should be placed on stimulating persons diagnosed with the disease. In light of how the illness evolves, it is important, also, to ensure a smooth transition in the services offered these persons and their caregivers. Young people could take part in support groups specifically designed for them in the early stages of the illness. Support groups could also be organized for caregivers of like age going through similar situations.

## Conclusion

This study revealed that many early-onset dementia caregivers had unmet support needs and required psycho-educational interventions to help them cope with the intangible aspects of their caregiver role, particularly changes in their marital, family and professional lives. Aside from wanting to be heard, these caregivers underscored the importance of receiving support in a timely fashion, taking account of their specific needs across their singular, less documented trajectory. In sum, as actors of their own reality wanting to take part in the decisions concerning them, the caregivers clearly recommended avoiding the “one-size-fits-all” approach to intervention and instead developing interventions tailored to their needs. Members of interprofessional teams who work with these family caregivers would stand to benefit from hearing them out knowing that in the absence of such a fit, caregivers are likely to perceive services as irrelevant and either reject them or accept them reluctantly and thus dampen their potential benefits [17].
